# Supplementary material for: Senescent cells limit p53 activity via multiple mechanisms to remain viable
Source: Nat Commun. 2022 Jun 28;13:3722. doi: 10.1038/s41467-022-31239-x (PMC9240076; doi:10.1038/s41467-022-31239-x)

SUPPLEMENTARY INFORMATION

Senescent cells limit p53 activity via multiple mechanisms to remain viable

Ines Sturmlechner[1,2], Chance C. Sine[1], Karthik B. Jeganathan[1], Cheng Zhang[3], Raul O. Fierro Velasco[1], Darren J. Baker[1,4], Hu Li[3] and Jan M. van Deursen[1,4,*]

[1]Department of Pediatric and Adolescent Medicine, Mayo Clinic, Rochester MN, United States, [2]Department of Pediatrics, Molecular Genetics Section, University of Groningen, University Medical Center Groningen, Groningen, The Netherlands, [3]Department of Molecular Pharmacology and Experimental Therapeutics, Mayo Clinic, Rochester, MN, United States, and [4]Department of Biochemistry and Molecular Biology, Mayo Clinic, Rochester MN, United States. *Correspondence: janvandeursen2@gmail.com

TABLE OF CONTENTS

Supplementary Figures 1-11
Supplementary Tables 1-2
Uncropped blots for Supplementary Figures

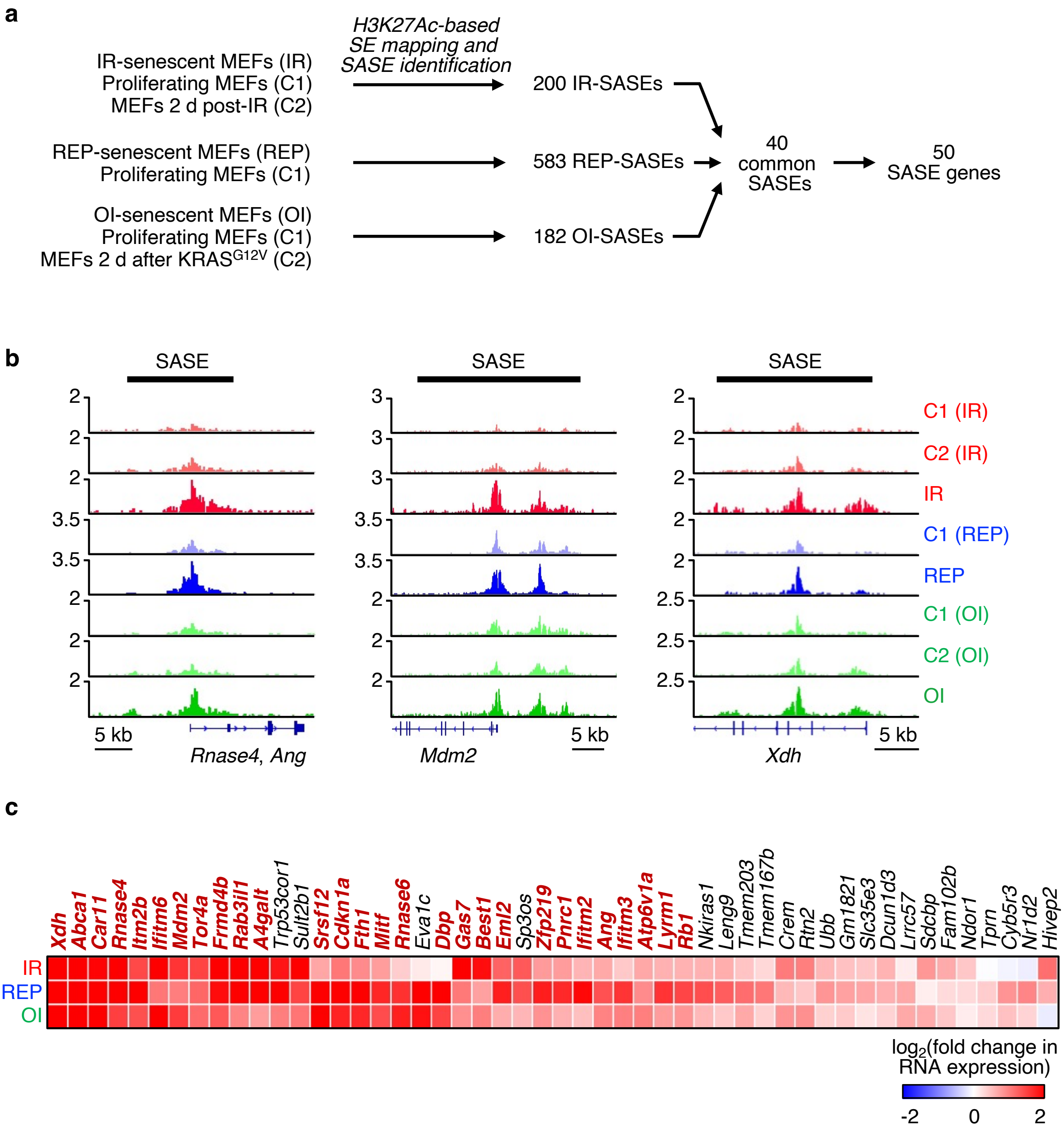

Supplementary Figure 1. Identification of senescence-associated super-enhancer genes. **a** Overview of senescence-associated super-enhancer (SASE) identification as well as genes that are transcriptionally activated in the vicinity of SASEs in senescent MEFs. Three distinct senescence-inducing stimuli were assessed: high-dose γ-irradiation (IR), extensive replication (REP), or oncogenic signaling by overexpression of KRAS^{G12V} (OI). Non-senescent (C1) and stressed, non-senescent cell cultures 2 days after IR or KRAS^{G12V} (C2) served as controls. **b** Representative H3K27Ac occupancy plots of three SASE loci in the indicated MEF cultures. Black bars denote SASE location. Y-axes depict cpm (counts per million mapped reads). **c** Heatmap showing log$_2$ fold change gene expression of 50 candidate SASE genes as measured by RNA-seq of SNCs versus proliferating counterparts. Red bolded genes were selected for follow up experiments in this study. Data are based on previously published ChIP-seq and RNA-seq experiments and are documented in GSE117278. Two to three independent MEF lines were used for ChIP-seq experiments and three independent MEF lines for RNA-seq.

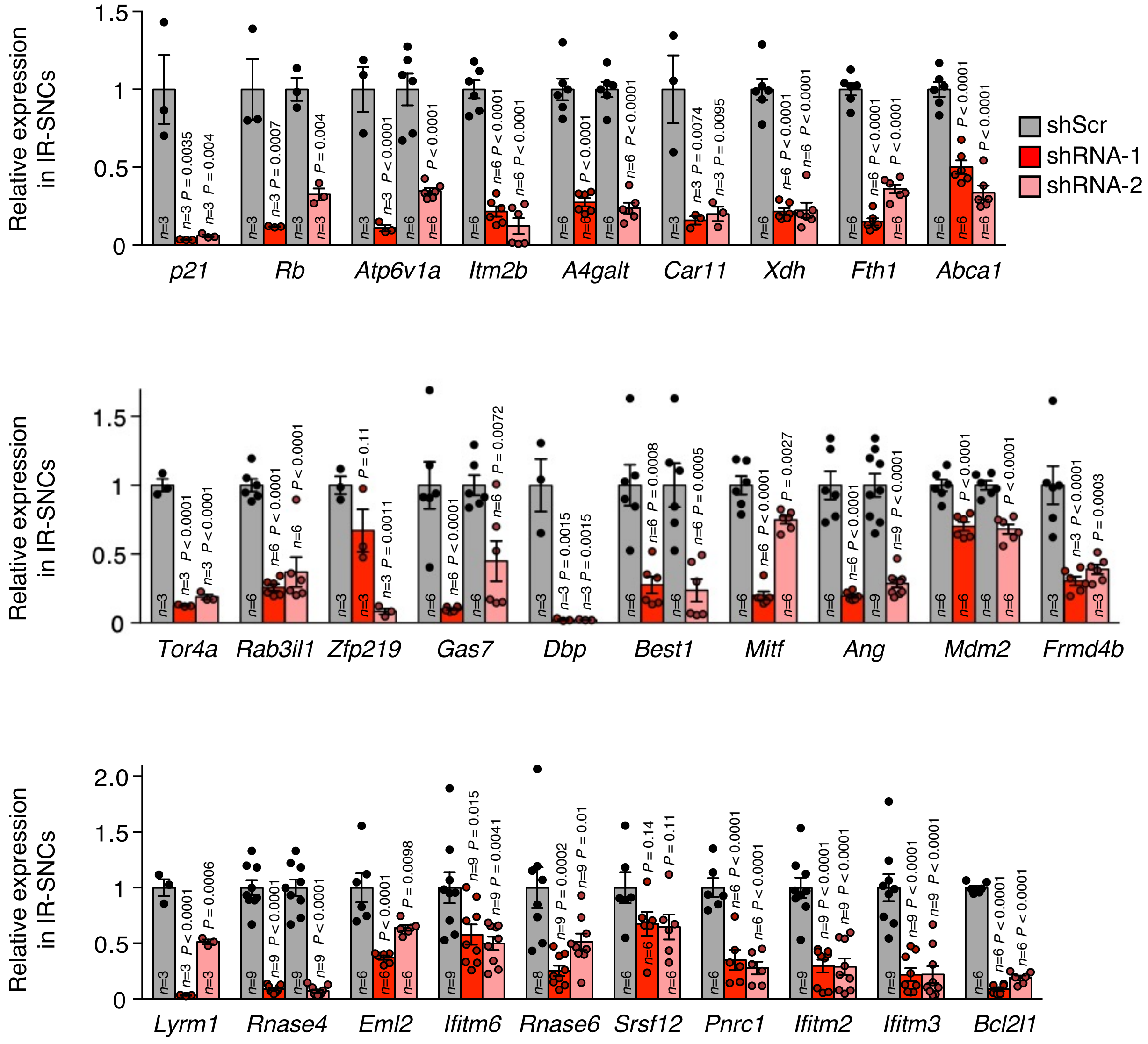

Supplementary Figure 2. SASE gene shRNA knockdown efficiency in IR-SNCs. RT-qPCR-based assessment of SASE gene expression in IR-senescent MEFs 3 days after infection with lentiviruses expressing the indicated shRNAs. We note that due to the experimental setup some *shScr* control values were used for comparisons of both shRNAs when they were assessed in the same experiment. Data represent means ± SEM. *n* depict independent MEF lines that were pooled from 1 to 3 independent experiments. Statistics: one-way ANOVA with Sidak's correction. Source data are provided as a Source Data file.

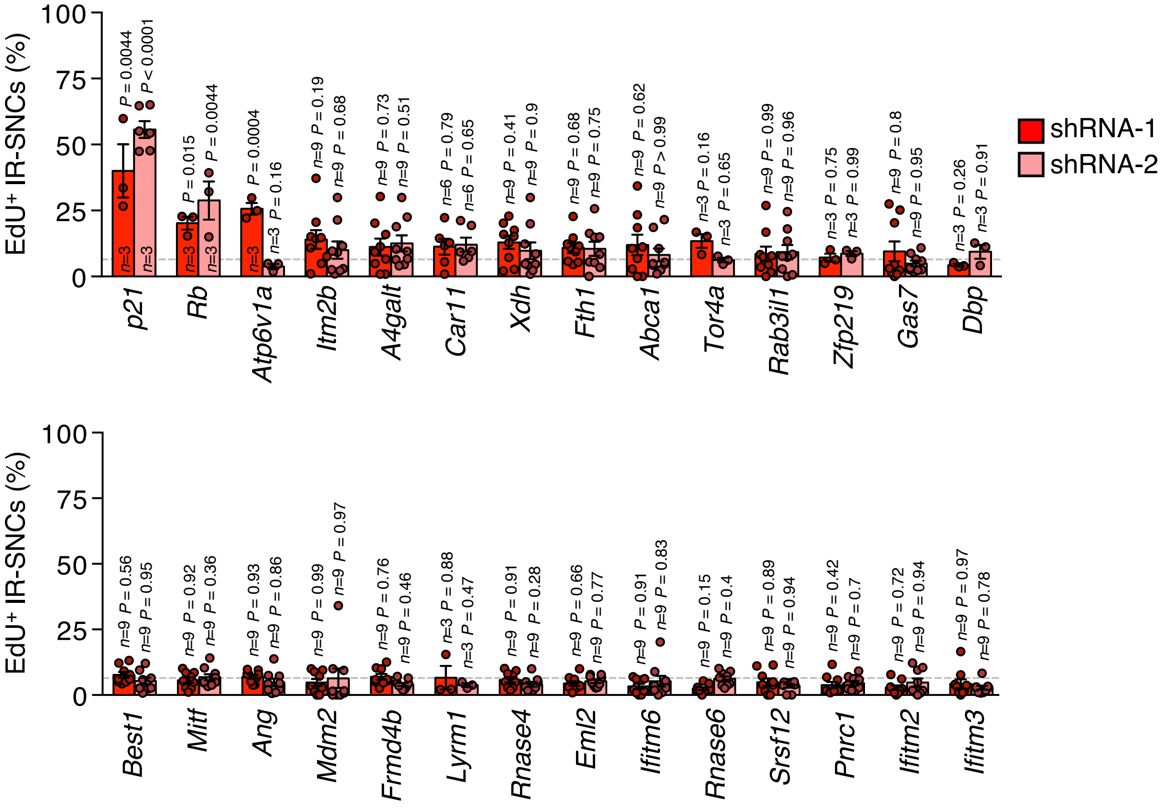

Supplementary Figure 3. SNC cycle arrest is controlled by few SASE genes. Percentage EdU-positive IR-SNCs at day 4 after SASE gene knockdown with two independent shRNAs per gene. Cells were allowed to incorporate EdU into their DNA for the last 2 days. Baseline value for *shScr* SNCs was experiment-specific; average of all *shScr*-infected MEF lines was 6.45% (line). Data represent means ± SEM. *n* depict independent MEF lines that were pooled from 1 to 3 independent experiments. Statistics: one-way ANOVA with Sidak's correction. Source data are provided as a Source Data file.

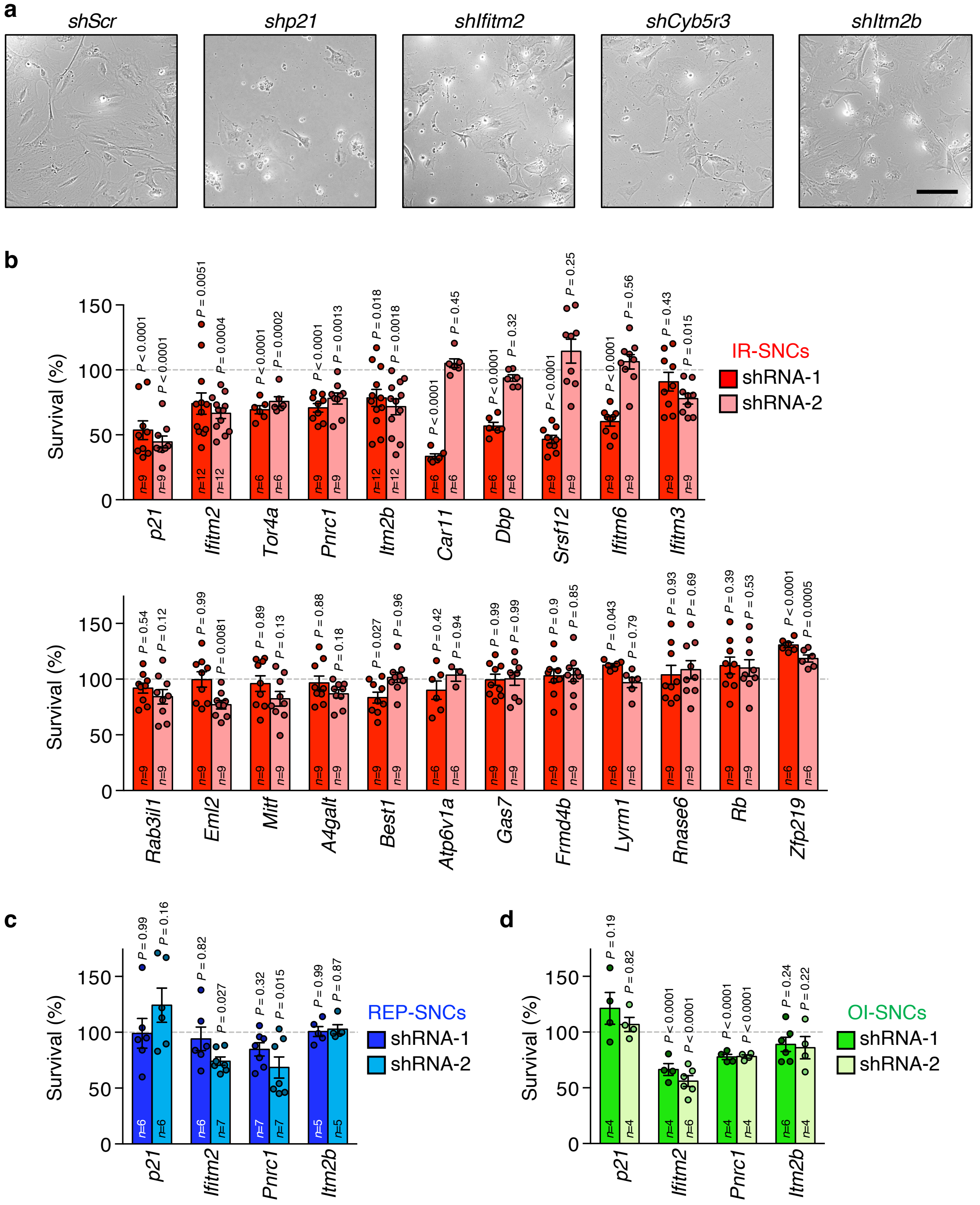

Supplementary Figure 4. Some SASE genes support survival of a subset of SNC types. **a** Representative images of IR-senescent MEFs 6 days after shRNA-mediated knockdown of the indicated SASE genes. **b-d** Survival of IR- (**b**), REP- (**c**) and OI-SNCs (**d**) at day 6 after SASE gene knockdown with two independent shRNAs per gene. Survival was measured by MTS assay. Comparisons were made to SNCs of same cultures infected with scrambled shRNA (*shScr*; 100% line). Images are representative of 3 independent experiments in **a**. Scale bar, 200 μm. Data represent means ± SEM. *n* depict independent MEF lines that were pooled from 1 to 4 independent experiments in **b**, 3 to 4 independent experiments in **c**,**d**. Statistics: one-way ANOVA with Sidak's correction in **b-d**. Source data are provided as a Source Data file.

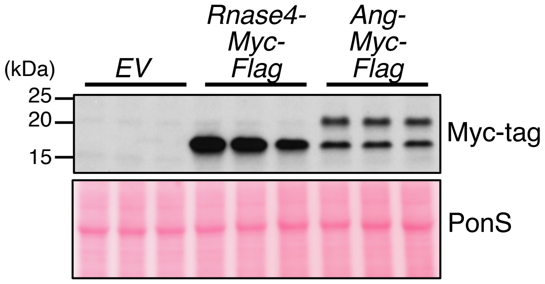

Supplementary Figure 5. Ectopic expression of tagged RNASE4 and ANG. Western blot of MEF lysates 3 days after infection with viruses containing Myc-Flag-tagged *Rnase4* or *Ang* cDNA or empty vector (*EV*). Ponceau S (PonS) served as loading control. Western blot results are representative of 2 independent experiments. Source data are provided as a Source Data file.

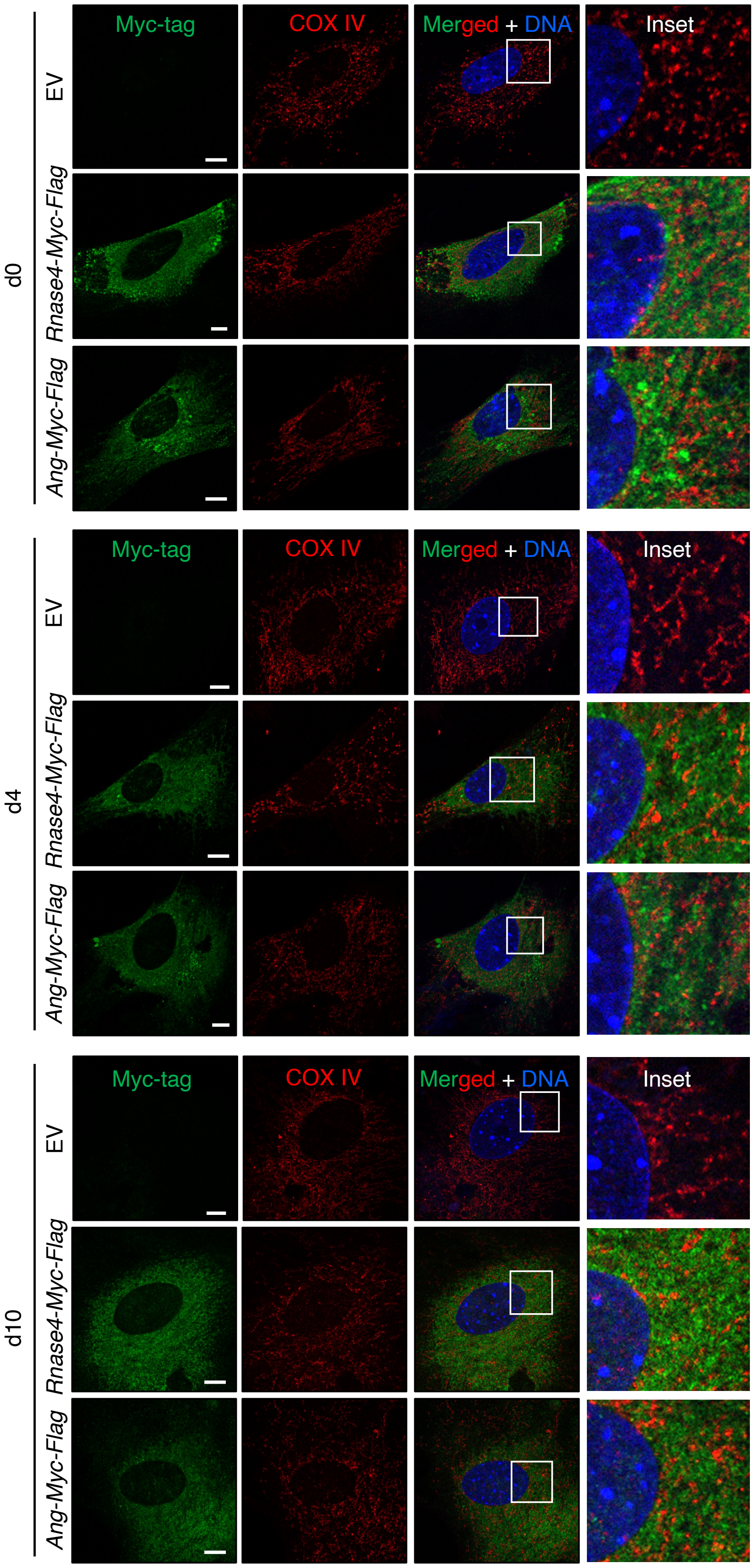
Myc-tag
COX IV
Merged + DNA
Inset
d0
EV
Rnase4-Myc-Flag
Ang-Myc-Flag
Myc-tag
COX IV
Merged + DNA
Inset
d4
EV
Rnase4-Myc-Flag
Ang-Myc-Flag
Myc-tag
COX IV
Merged + DNA
Inset
d10
EV
Rnase4-Myc-Flag
Ang-Myc-Flag

Supplementary Figure 6. RNASE4 and ANG show cytoplasmic localization without overt mitochondrial accumulation. Representative immunofluorescence images of MEFs infected with indicated constructs at indicated times post-irradiation. Cells were stained for Myc-Flag-tagged RNASE4 or ANG using a Myc-tag antibody and mitochondria were visualized via COX IV staining. Images are representative of 2 independent experiments. Scale bars, 10 μm.

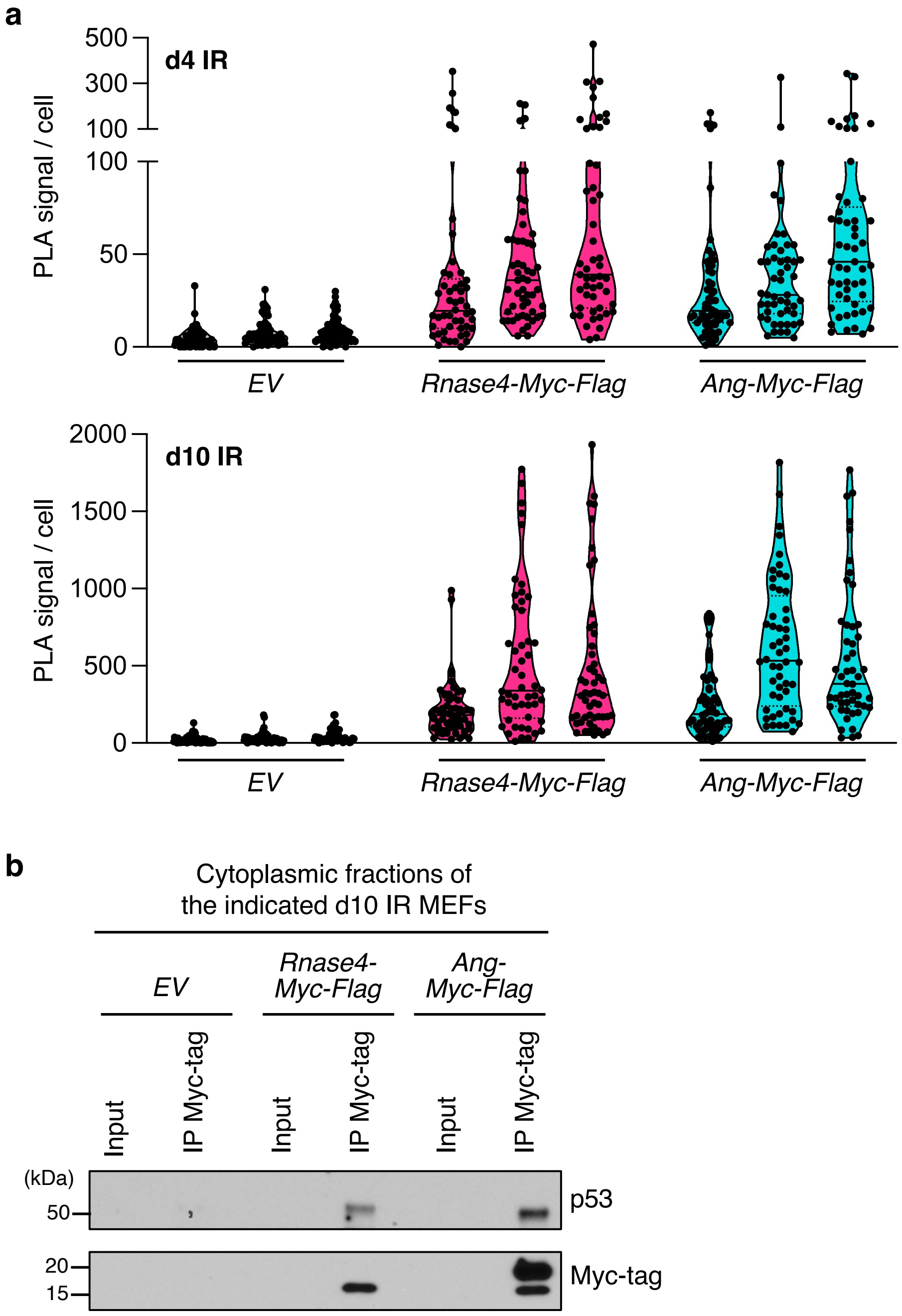

Supplementary Figure 7. Analysis of RNASE4-p53 and ANG-p53 complex formation. a. Violin blots showing distribution of proximity ligation assay (PLA) signals in indicated conditions and timepoints post-irradiation. PLA was assessed between endogenous p53 and Myc-Flag-tagged RNASE4 or ANG. PLA signals per cell per independent MEF line are depicted. **b.** Cytosolic fractions of MEFs stably expressing Myc-Flag-tagged RNASE4 or ANG, subjected to immunoprecipitation with Myc-tag-specific antibodies, and analyzed for co-precipitation of p53. One experiment was performed in **b**. Data in **a** correspond to data shown in Fig. 7**c**,**d**. Source data are provided as a Source Data file.

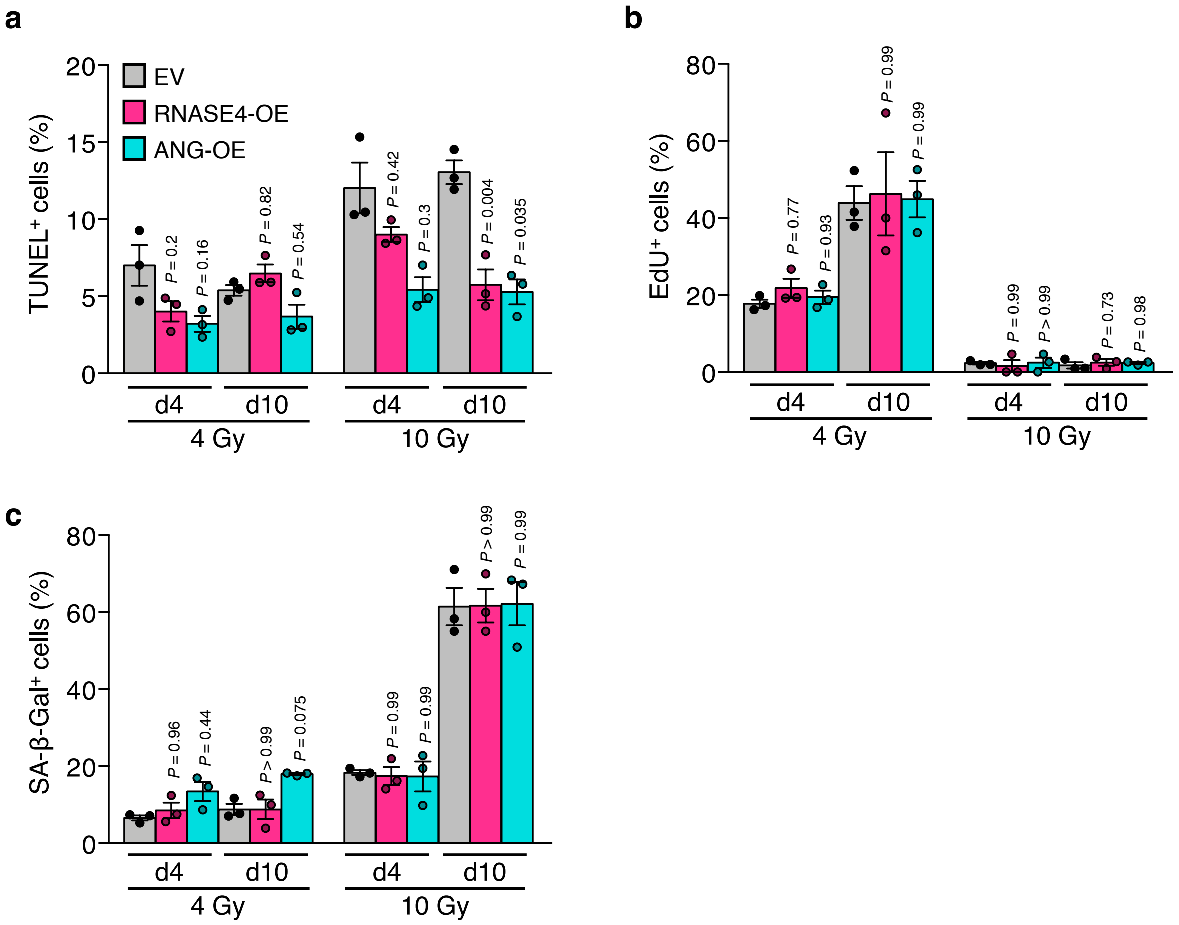

Supplementary Figure 8. RNASE4 or ANG overexpression largely preserves cell fate decisions of stressed cells. a TUNEL assay to assess percentage of dying cells. **b** Proportion of EdU-positive cells in indicated conditions and timepoints post-irradiation in MEFs overexpressing Myc-Flag-tagged RNASE4 or ANG. Cells were allowed to incorporate EdU for 24 hours. **c** Quantification of MEFs positive for senescence-associated β-galactosidase (SA-β-Gal). 4 Gy and 10 Gy indicate amount of irradiation. Data represent means ± SEM. n = 3 independent MEF lines from 1 experiment in **a**-**c**. Statistics: one-way ANOVA with Sidak's correction in **a**-**c**. Source data are provided as a Source Data file.

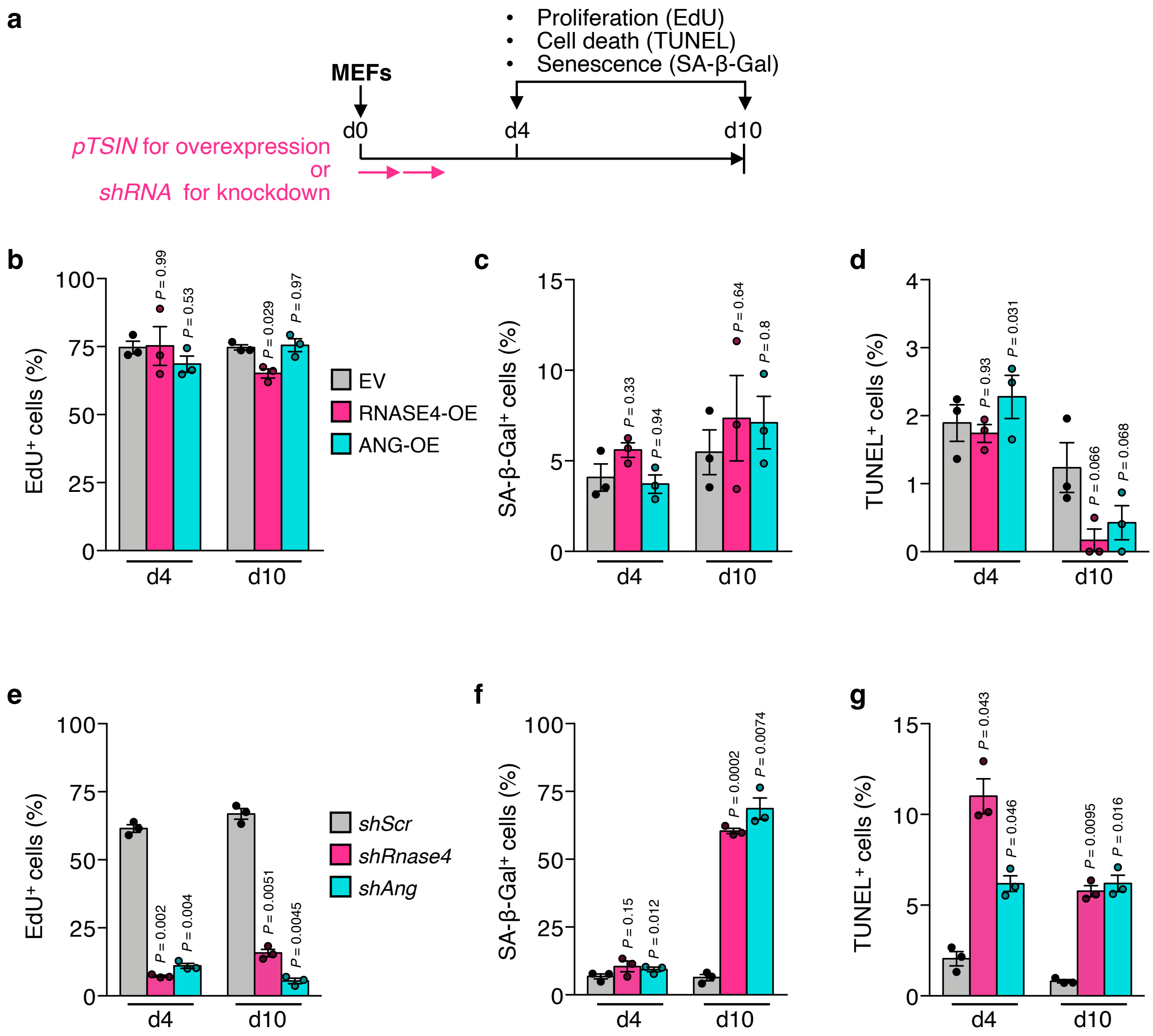

Supplementary Figure 9. RNASE4 or ANG depletion is sufficient to induce cell cycle arrest and senescence. a Experimental approach to assess indicated cell fates after *Rnase4* or *Ang* overexpression or knockdown in MEFs in the absence of stress. **b** Quantification of EdU-positive cells after gene overexpression. Cells were allowed to incorporate EdU into the DNA for 24 hours. **c** Proportion of cells positive for senescence-associated β-galactosidase after gene overexpression. **d** TUNEL assay to assess the proportion of dying cells after gene overexpression. **e** as in **b** but after knockdown of *Rnase4* or *Ang*. **f** as in **c** but after knockdown of *Rnase4* or *Ang*. **g** as in **d** but after knockdown of *Rnase4* or *Ang*. Data represent means ± SEM. *n* = 3 independent MEF lines from 1 experiment in **b-g**. Statistics: one-way ANOVA with Sidak's correction in **b-g**. Source data are provided as a Source Data file.

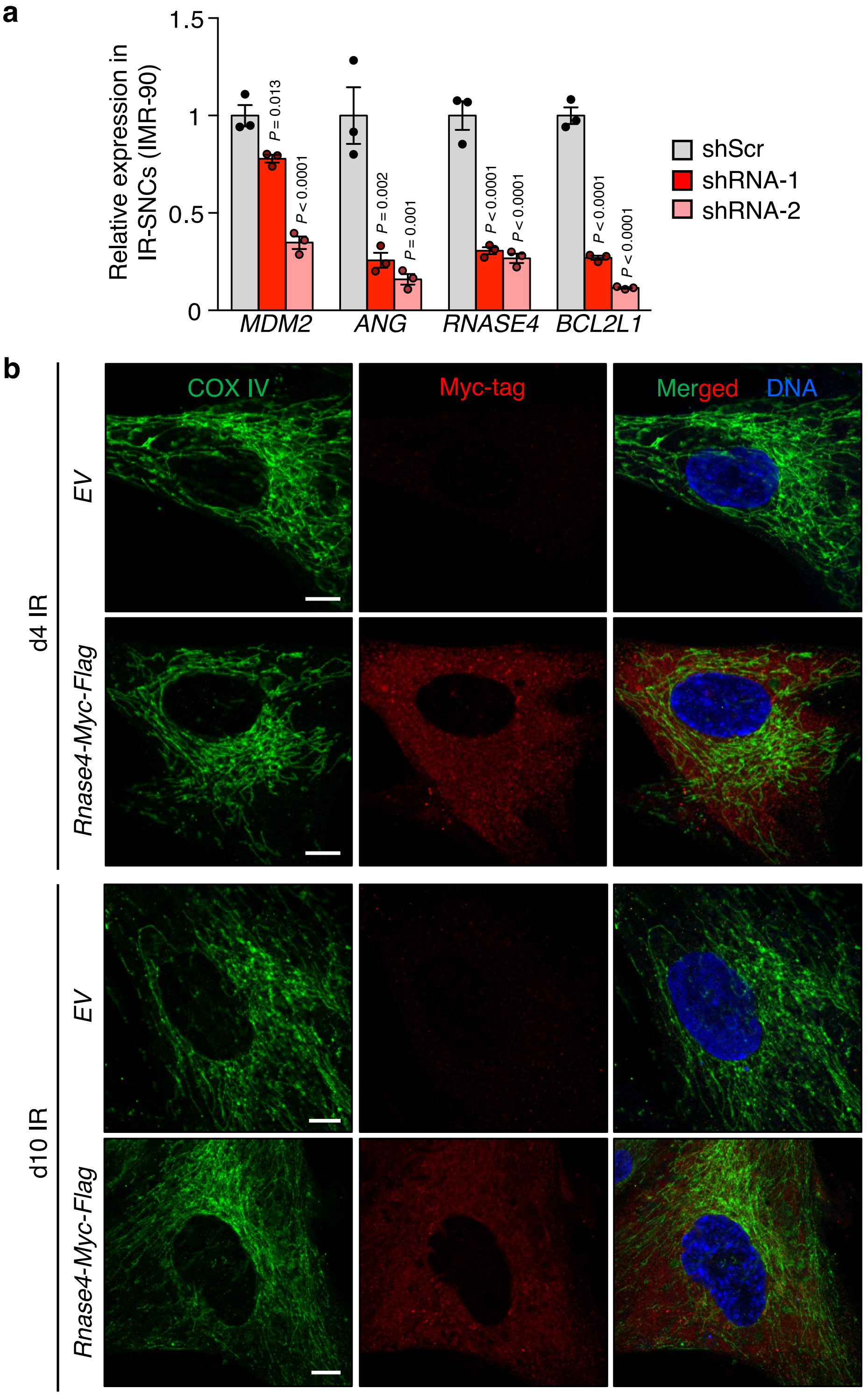

Supplementary Figure 10. RNASE4 localizes predominantly to the soluble cytoplasmic fraction in IMR-90 cells. a RT-qPCR-based assessment of SASE gene knockdown in IR-senescent IMR-90 cells 3 days after infection with lentiviruses expressing the indicated shRNAs. **b** Representative immunofluorescence images of IMR-90 cells infected with *Rnase4*-overexpression construct or empty vector (*EV*) at indicated times post-irradiation. Cells were stained for Myc-Flag-tagged RNASE4 using a Myc-tag antibody and mitochondria were visualized via COX IV staining. Scale bars, 10 μm. Data represent means ± SEM. *n* = 3 technical IMR-90 replicates from 1 experiment in **a**. Statistics: one-way ANOVA with Sidak's correction in **a**. Source data are provided as a Source Data file.

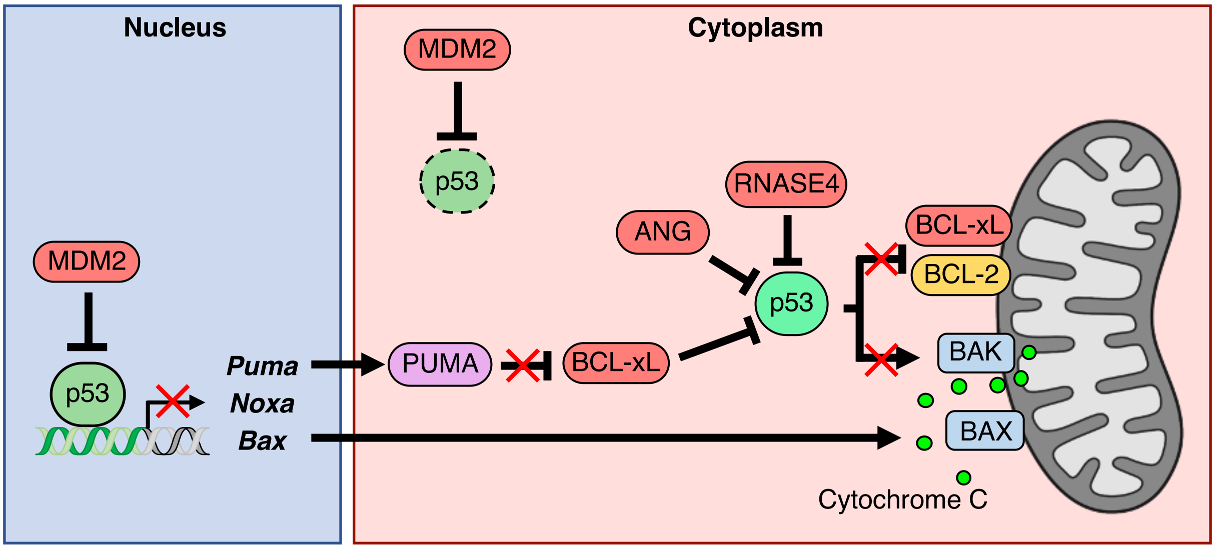

Supplementary Figure 11. Model of SASE gene-mediated suppression of p53 hyperactivity and pro-apoptotic functions in SNCs. SNCs uncouple p53 from p21 allowing for increased levels of p21 and its function in SNC core properties, while several other SASE-controlled genes act to limit p53 activity through multiple mechanisms. Of these, MDM2 and ANG keep the transcriptional activity of p53 in check to inhibit the expression of pro-apoptotic genes. ANG interacts with p53 in the cytoplasm, implying that it acts to inhibit p53 transcriptional activity through a cytoplasmic tethering mechanism. RNASE4 also binds to p53 in the cytoplasm and may exert its inhibitory effect through tethering as well, but now, like Bcl-xL, impacting p53's non-transcriptional pro-apoptotic functions involving BAK and BAX oligomerization and cytochrome c release. Extended studies indicate that ANG and RNASE4 also bind to cytoplasmic p53 in response to DNA damage in non-senescent cells, where they act to inhibit apoptosis and cellular senescence. Part of the figure was created via BioRender.

		target sequence (shRNA-1)	target sequence (shRNA-2)
mus musculus	***A4galt***	CACGCCATGCACTCGTTAGGT	GTGCAGACGTTTGCAGACAAA
	Abca1	GGCAAGGCCGCACCATTATTT	**GAGTGCCACTTTCCGAATAAA**
	Ang	GCCCGTTGTTCTTGATCTTCG	**TGGCAACAAGAGCAACATCAA**
	Atp6v1a	TAAGATCACATGGTCCATTAT	GCCACCATTCAGGTGTATGAA
	Bcl-xL (Bcl2l1)	**GTGGATCTCTACGGGAACAAT**	AGCTGGAGTCAGTTTAGTGAT
	Best1	CGTGGACTCTACAGAATGGTT	CGACTGGATAAGTATCCCATT
	Car11	GAACACCAGATCAACCATGAA	CCGGGACACTATCACCCGAAT
	Crem	GCCTGGTATTCCCAAGATTGA	CCTGGTATTCCCAAGATTGAA
	Cyb5r3	CCTTCCTATTGGCCAACACAT	GTGAAGTCTGTAGGCATGATT
	Dbp	AGAGGTCGAGAGATGCAAGAA	GCTCAGACTTACACCTGACTT
	Dcun1d4	ATGAGCGGGAGGATGCAATTT	GCGGTCACTGCATCGTGAAAT
	Eml2	GCTATCCACACAGATGGCAAT	CTAAAGCTAGACTGGGTGTAT
	Frmd4b	CCAGTATGACTTACAAGACAA	CGGTTACTATATCGCTGGATA
	Fth1	**CCTACGTCTATCTGTCTATGT**	CTATCTGTCTATGTCTTGTTA
	Gas7	GCTGGAGATCAAGTTGAGCAA	CCCAGTATTTCCAGGCCATAA
	Hivep2	GTCGCCTCTGAGGACCTATTT	GCCGTAAACCATACAAGAAAT
	Ifitm2	TGGTCTGGTCCCTGTTCAATA	ACTGCCAAGTGCCTGAATATC
	Ifitm3	ACTACGAAAGAATCAAGGAAG	CTCAGCATCCTGATGGTTGTT
	Ifitm6	GTCTACATTTAACACAGTGTT	GCCAAGATTCTGAACATCCTT
	Itm2b	GGGCGTACCTGTACAAGTATT	CCTCAGTCCTACCTTATCCAT
	Lrrc57	CCTTTGTCAAAGGAGCACCAA	CATCGATTTATCCAACAACAA
	Lyrm1	CGGATGCAAAGGGATTAGCTA	CCAAACCAGTGTATCTGAAGT
	Mdm2	**GTGTACCTCATGCAATGAAAT**	CCAATCCAAATGATTGTGCTA
	Mitf	GCCAGACTTGTATATTCTATT	TCAGCCTGGAATCAAGTTATA
	Nkiras	CACTGATTGAACCCTTCACTT	GAGCAACTCCTTTATGGGAAT
	p21 (Cdkn1a)	GACCAGCCTGACAGATTTCTA	CTATCACTCCAAGCGCAGATT
	p53	CCAGTCTACTTCCCGCCATAA	
	Pnrc1	ATTACGCCGGAGCAAAGTTCA	ATACAGTGGAGATAGTCTAAG
	Rab3il1	GTATGCAACTTCTTCACCTAT	CTTCTTCACCTATATTCGCTA
	Rb	CGCTATGAAGAAGTTTATCTT	CCGTGGATTCTGAACGTACTT
	Rnase4	**CATCTGCAGTACCACCAATAT**	CGTGCCTTATACTCACATATA
	Rnase6	GCACAATATCACCTGCAAGAA	CCTGTACACTTAGATAAGATT
	Rtn2	GTTGAGCCACATCAAAGCTAA	GCTGGGACTTGTAGTTGAATA
	Sdcbp	GATGGAAATCTGTATCCTAAA	GCAGTGGACATGTTGGCTTTA
	Slc35e3	CCTCACCTACACCCACTTTAA	GAAGAGATTCTCCGTCAGAAT
	Srsf12	ATACAGTGGAGATAGTCTAAG	GCCAGCTTGTGCATGGTATAC
	Tor4a	CGACTAAGTGAGGGTACAAAC	ATTGAGAATCTAGACGATAAT
	Xdh	**GCTTGAATCCTGCCATTGATA**	GCTGTGTATAATCCCGACTAA
	Zfp219	CTGGTTTCTCAAGGGTCACAT	GAAAGCGCTTCCGATTCAATT
homo sapiens	***ANG***	AGAATAAGCAAGTCTTCTTTC	ACGTTGTTGTTGCTTGTGAAA
	BCL-xL (BCL2L1)	GTGGATCTCTACGGGAACAAT	AGCTGGAGTCAGTTTAGTGAT
	MDM2	GTGTACCTCATGCAATGAAAT	**GATTCCAGAGAGTCATGTGTT**
	RNASE4	**CATCTGCAGTACCACCAATAT**	CGTGCCTTATACTCACATATA
	P53	GACTCCAGTGGTAATCTAC	

Supplementary Table 1. Sequences of shRNAs used for gene knockdown. Bolded sequence indicates shRNA used for mechanistic experiments in which one hairpin was used.

		Primer forward (5'-3')	Primer reverse (5'-3')
mus musculus	***A4galt***	CTAACCCAGTGTGCATGTCC	GTCTCCTCCAGATGGGAACA
	Abca1	CATGCACAAGGTCCTGAGAA	GGAAGGGACAAATTGTGCTG
	Ang	CCAGGCCCGTTGTTCTTGAT	GGAAGGGAGACTTGCTCATTC
	Atp6v1a	ACTTTCTGCATTTCGAGCCC	TCACAGGCTGTAACCACAGG
	Bax	CAGGATGCGTCCACCAAGAA	AGTCCGTGTCCACGTCAGCA
	Bcl-xL (Bcl2l1)	GTTGGATGGCCACCTATCTG	GCTGCATTGTTCCCGTAGAG
	Best1	AGTGTGGACAGCTGTATGCC	GTTCAGAAACTGCCTCCCGA
	Car11	GCTGAAGCGGGTTCTTTATG	CAGACACGTTGACCACAGGT
	Ccl2	ATTGGGATCATCTTGCTGGT	CCTGGTGTTCACAGTTGCC
	Crem	AGGCAAATGACCATGGAAAC	TCTGTCTCTGCAATTGTTGCT
	Cxcl1	GTGCCATCAGAGCAGTCTGT	ACCCAAACCGAAGTCATAGC
	Cyb5r3	TAGCGGGTGTAGAACCGTG	TGTAGATGAACCAGACGGGC
	Dbp	CTGGCCCGAGTCTTTTTGC	CCAGGTCCACGTATTCCACG
	Dcun1d3	GGTTCCCTAGCCTCTTAACAGA	ACTGGTCCAATACTGGAGGATT
	Eml2	GGTTCGGACTACAGCAAGGT	CCACGTACAGTGTGTCTCCC
	Frmd4b	CACCTGCTGGATGACAGAAGA	ACCAGTTCTCTTGACCCGTG
	Fth1	CATCAACCGCCAGATCAAC	GGCAAAGTTCTTCAGAGCCA
	Gas7	CCCTTCCCTCAACCTCTTTC	CCACCCCTGAAGTCTTTGAA
	Hivep2	CGTCTGGAAGCAGTTTGCAC	ATACTTTCCCCGTCCACGTC
	Ifitm2	GCTTCGTTGCCTATGCCTAC	AGGGAGCTGATATTCAGGCA
	Ifitm3	AGCCTATGCCTACTCCGTGA	GAGGACCAAGGTGCTGATGT
	Ifitm6	TGCCTGGGTTTCATTGCCTA	GTTGAAGCATGGGATTGGGC
	Itm2b	AAGAAGGACGAGCCCAAGAG	CTCCTCTGTCCAACCGGAAC
	Killer (Tnfrsf10b)	CGGGCAGATCACTACACCC	TGTTACTGGAACAAAGACAGCC
	Lrrc57	CTCTCCGTGCTCATGTGGAAA	GGGGAACTCGGTCAGACCA
	Lyrm1	GCAGCAACCCGACAAGAAGT	CGGGCCGTGCATTCAACTAT
	Mdm2	TCTGTGAAGGAGCACAGGAA	TCCTTCAGATCACTCCCACC
	Mitf	GAAATTTTGGGCTTGATGGA	GGAGTTGCTGATGGTAAGGC
	Mmp2	TGCAGGAGACAAGTTCTGGA	GACGGCATCCAGGTTATCAG
	Nkiras	GTTGGGAAAACTGCAATTTTGGA	ACCCCACGGTCTGTCTCTAC
	Noxa (Pmaip1)	GAAGTCGCAAAAGAGCAGGA	ACTTTGTCTCCAATCCTCCG
	Pnrc1	CACTCGCGAAGGAGGTTTTA	GCAGAATCCTGCCAAAAGTC
	Puma (Bbc3)	ACGACCTCAACGCGCAGTACG	GAGGAGTCCCATGAAGAGATTG
	Rab3il1	AGACGCTGGGATGTGGAG	GTTACTTTTGCTGGGGCCTA
	Rb	GAACAGATTTGTCCTTCCCG	CCATGATTCGATGCTCACAT
	Rnase4	CTTCTGTCCAGTGCAAACGC	CTGTCTCTCTGCAGTCCGTG
	Rnase6	GATCATCTGGCCCTGTTCAC	CCACAGCTCAAGCAGCAATA
	Rtn2	CAGAATTCATGTTGGAGCCG	CAGAGGAGAGAGGCCATGAG
	Sdcbp	CAGGCGTTTGGAGAGAAGAT	CCAACATGTCCACTGCTGTC
	Slc35e3	TTTGGTCCCTGGTCAGTTTC	GCAGAACTTGAAGTGTCCGAA
	Srsf12	AACACCTCCCTGTTCGTCAG	GGATCTCCTGTGGTCACTCG
	Tbp	GGCCTCTCAGAAGCATCACTA	GCCAAGCCCTGAGCATAA
	Tnf	CAGCCTCTTCTCATTCCTGC	AGGGTCTGGGCCATAGAACT
	Tor4a	CTACGTGCCGACCTAGACCA	TTGCGGGGTGTCCTAGAAGT
	Xdh	GGTTGTTTCCACTTCCTCCA	CGAAACAAGCCTGAGCCTAC
	Zfp219	AAGAAGTGGTGGAGGCTGAA	GCGGAAAGATTTTCCACAGA
homo sapiens	***ANG***	CAAGGCCATCTGTGAAAACAAG	CAGGGGGAACCTCCATGTAG
	BCL-xL (BCL2L1)	TGAATGACCACCTAGAGCCTT	GAAGAGTGAGCCCAGCAGAAC
	MDM2	CAGTAGCAGTGAATCTACAGGGA	CTGATCCAACCAATCACCTGAAT
	RNASE4	TGCAGAGGACCCATTCATTGC	TCAAGTTGCAGTAGCGATCAC
	TBP	GCCAGCTTCGGAGAGTTCTGGGATT	CGGGCACGAAGTGCAATGGTCTTTA

Supplementary Table 2. Primer sequences for RT-qPCR analyses.

Uncropped blots: Supplementary Figure 5

Myc-tag

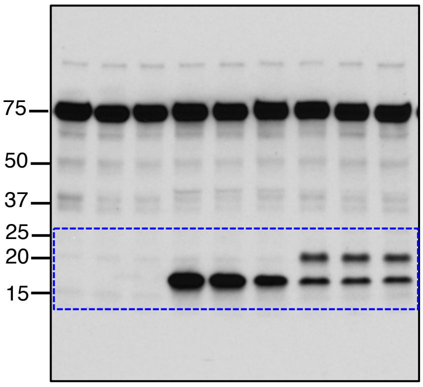

Ponceau S

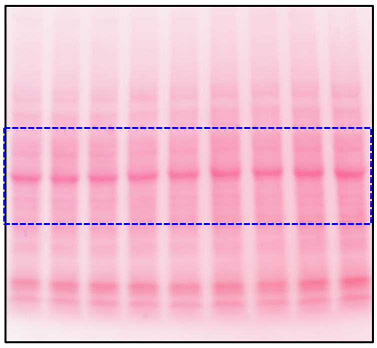

Uncropped blots: Supplementary Figure 7b

p53

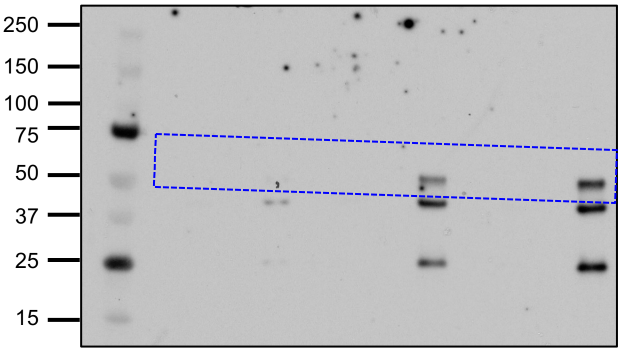

Myc Tag

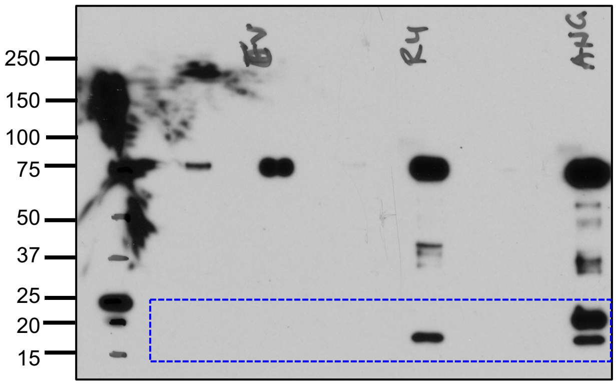

Supplement: Supplementary file 1 — Supplementary Information [file 41467_2022_31239_MOESM1_ESM.pdf]
