# Supplementary material for: Senescent cells limit p53 activity via multiple mechanisms to remain viable
Source: Nat Commun. 2022 Jun 28;13:3722. doi: 10.1038/s41467-022-31239-x (PMC9240076; doi:10.1038/s41467-022-31239-x)
Supplement: Supplementary file 3 — Reporting Summary [file 41467_2022_31239_MOESM3_ESM.pdf]

nature portfolio

Corresponding author(s): Jan M. van Deursen

Last updated by author(s): Apr 22, 2022

Reporting Summary

Nature Portfolio wishes to improve the reproducibility of the work that we publish. This form provides structure for consistency and transparency in reporting. For further information on Nature Portfolio policies, see our Editorial Policies and the Editorial Policy Checklist.

Statistics

For all statistical analyses, confirm that the following items are present in the figure legend, table legend, main text, or Methods section.

n/a	Confirmed	
☐	☒	The exact sample size (*n*) for each experimental group/condition, given as a discrete number and unit of measurement
☐	☒	A statement on whether measurements were taken from distinct samples or whether the same sample was measured repeatedly
☐	☒	The statistical test(s) used AND whether they are one- or two-sided<br>*Only common tests should be described solely by name; describe more complex techniques in the Methods section.*
☒	☐	A description of all covariates tested
☐	☒	A description of any assumptions or corrections, such as tests of normality and adjustment for multiple comparisons
☐	☒	A full description of the statistical parameters including central tendency (e.g. means) or other basic estimates (e.g. regression coefficient) AND variation (e.g. standard deviation) or associated estimates of uncertainty (e.g. confidence intervals)
☐	☒	For null hypothesis testing, the test statistic (e.g. *F*, *t*, *r*) with confidence intervals, effect sizes, degrees of freedom and *P* value noted<br>*Give P values as exact values whenever suitable.*
☒	☐	For Bayesian analysis, information on the choice of priors and Markov chain Monte Carlo settings
☒	☐	For hierarchical and complex designs, identification of the appropriate level for tests and full reporting of outcomes
☒	☐	Estimates of effect sizes (e.g. Cohen's *d*, Pearson's *r*), indicating how they were calculated

Our web collection on statistics for biologists contains articles on many of the points above.

Software and code

Policy information about availability of computer code

Data collection	Zeiss ZEN software was used to acquire images of immunofluorescence experiments on a Zeiss LSM880 confocal laser-scanning microscope. Olympus cellSens 2.3 was used to acquire fluorescence and DIC images for the following assays: TUNEL, EdU, SAbGal, cell morphology.
Data analysis	GraphPad Prism 6.0 and 9.0 was used for statistical assessments. ImageJ 2.1.0 was used for image quantifications of proximity ligation assays and immunoblot densitometry. Zeiss ZEN software was used to export immunofluorescence images. Quantstudio 6 (Applied Biosystems) was used for RT-qPCR and RT-qPCR analysis. IGV genome browser 2.10.2 was used the generate H3K27ac peak profiles. Morpheus (Broad Institute) was used to generate heatmaps.

For manuscripts utilizing custom algorithms or software that are central to the research but not yet described in published literature, software must be made available to editors and reviewers. We strongly encourage code deposition in a community repository (e.g. GitHub). See the Nature Portfolio guidelines for submitting code & software for further information.

Data

Policy information about availability of data

All manuscripts must include a data availability statement. This statement should provide the following information, where applicable:

- Accession codes, unique identifiers, or web links for publicly available datasets
- A description of any restrictions on data availability
- For clinical datasets or third party data, please ensure that the statement adheres to our policy

ChIP-seq and RNA-seq data sets have been previously deposited in the Gene Expression Omnibus under accession number GSE117278.

Field-specific reporting

Please select the one below that is the best fit for your research. If you are not sure, read the appropriate sections before making your selection.

- [x] Life sciences
- [ ] Behavioural & social sciences
- [ ] Ecological, evolutionary & environmental sciences

For a reference copy of the document with all sections, see nature.com/documents/nr-reporting-summary-flat.pdf

Life sciences study design

All studies must disclose on these points even when the disclosure is negative.

Sample size

Sample sizes were based on previously published experiments where differences were observed. We note that no power calculations were used. The following previously published studies guided our samples sizes used for cell line-based work:
-) Sturmlechner et al. 2021 (PMID 34709885) p21 produces a bioactive secretome that places stressed cells under immunosurveillance.
-) Sieben et al. 2020 (PMID 31738183) BubR1 allelic effects drive phenotypic heterogeneity in mosaic-variegated aneuploidy progeria syndrome.
-) Baker et al. 2004 (PMID 15208629) BubR1 insufficiency causes early onset of aging-associated phenotypes and infertility in mice.
-) Limzerwala et al 2020 (PMID 34841254) FoxM1 insufficiency hyperactivates Ect2-RhoA-mDia1 signaling to drive cancer.

Data exclusions

No samples were excluded from the study.

Replication

Number of replicates is described in figures or figure legends and it is indicated whether they were technical or biological replicates.

Randomization

Randomization was not applicable to any experiments performed due to lack of drug treatments or interventions as well as experiments needing clearly defined groups of mice or cell lines based on genotype.

Blinding

Blinding to allocation during experiments was not applicable to any of the experiments. Investigators were not blinded to assessment outcome during experimentation, as they were performed by the same researcher for consistency and feasibility.

Reporting for specific materials, systems and methods

We require information from authors about some types of materials, experimental systems and methods used in many studies. Here, indicate whether each material, system or method listed is relevant to your study. If you are not sure if a list item applies to your research, read the appropriate section before selecting a response.

Materials & experimental systems

n/a	Involved in the study	
☐	☒	Antibodies
☐	☒	Eukaryotic cell lines
☒	☐	Palaeontology and archaeology
☐	☒	Animals and other organisms
☒	☐	Human research participants
☒	☐	Clinical data
☒	☐	Dual use research of concern

Methods

n/a	Involved in the study	
☒	☐	ChIP-seq
☒	☐	Flow cytometry
☒	☐	MRI-based neuroimaging

Antibodies

Antibodies used

All antibodies used are listed in the Methods section.

Co-immunoprecipitation: mouse anti-Myc-tag (9B11, Cell Signaling, #2276 with 10 ul per IP or #5698 with 15 ul per IP)
Co-immunoprecipitation: mouse anti-FLAG (Sigma, #F3165; 1:1,000

Western blot: rabbit anti-Myc-tag (Cell Signaling, #2272, 1:1,000)
Western blot: mouse anti-p53 (Cell Signaling, #2524S; 1:1,000)
Western blot: mouse anti-p53-HRP (Santa Cruz, sc-126; 1:1,000)
Western blot: rabbit anti-phospho-P53 S15 (Cell Signaling, #9284; 1:1,000)
Western blot: mouse anti-p21 (Santa Cruz, sc-53870; 1:8,000)
Western blot: rabbit anti-p16 (Santa Cruz, sc-1207; 1:1,000)
Western blot: rabbit anti-BAX (Cell Signaling, #2772; 1:1,000)
Western blot: rabbit, anti-BAD (Cell Signaling, #9292; 1:1000)
Western blot: rabbit anti-BCL2 (Santa Cruz, sc-492; 1:1,000)
Western blot: mouse anti-BCL-xL (Santa Cruz, sc-8392; 1:1000)

Western blot: rabbit, anti-α-Tubulin (Cell Signaling, #2125; 1:1000)
Western blot: rabbit, anti-HDAC2 (Abcam, ab7029; 1:1000)
Western blot: rabbit, anti-EGFR (Cell Signaling, #71655; 1:1000)
Western blot: rabbit, anti-RNASE4 (generated by GenScript, see method section; 1:1000)
Western blot: goat anti-mouse, HRP-conjugated (Jackson Immunoresearch; #115-035-146; 1:10,000)
Western blot: goat anti-rabbit, HRP-conjugated (Jackson Immunoresearch; #111-035-003; 1:10,000)

Immunofluorescence: mouse, anti-COX IV (Abcam, ab33985; 1:100)
Immunofluorescence: rabbit, anti-Myc-tag (Cell Signaling, #2272, 1:100)
Immunofluorescence: goat, anti-mouse Alexa Fluor 488 (Invitrogen, #A11029; 1:250)
Immunofluorescence: goat, anti-rabbit Alexa Fluor 594 (Invitrogen, #A11012; 1:250)

Proximity ligation assay: mouse, anti-p53 (Cell Signaling, #2524S; 1:50)
Proximity ligation assay: rabbit, anti-Myc-tag (Cell Signaling, #2272, 1:100)

Validation

Commercially available ntibodies were validated by the manufacturer and the published literature utilizing these antibodies. If possible, antibodies were validated in house using gene knockout, gene knockdown or gene overexpression samples. Several antibodies used in this manuscript for Western Blot analysis, have accompanying knockdown data or treatment-mediated reductions or inductions of endogenous protein.

For previously employed antibodies, please see the following references:
Anti-Myc-tag, anti-p21, anti-p16 antibodies: Sturmlechner et al. 2021 (PMID 34709885)
Anti-p53, anti-phospho-P53 S15, anti-p21, anti-BAX, anti-HDAC2: Aziz et al. 2019 (PMID 30878468).
Anti-EGFR, anti-Tubulin, anti-HDAC2: Limzerwala et al. 2020 (PMID 34841254)

Please also find validation notes and citations for the utilized antibodies, directly on the websites of the manufacturers below.

mouse anti-Myc-tag (9B11, Cell Signaling, #2276 or #5698)
https://www.cellsignal.com/products/primary-antibodies/myc-tag-9b11-mouse-mab/2276

rabbit anti-Myc-tag (Cell Signaling, #2272)
https://www.cellsignal.com/products/primary-antibodies/myc-tag-antibody/2272?site-search-type=Products&N=4294956287&Ntt=%232272%29&fromPage=plp&_requestid=5815387

mouse anti-FLAG (Sigma, #F3165)
https://www.sigmaaldrich.com/US/en/product/sigma/f3165

mouse anti-p53 (Cell Signaling, #2524S) https://www.cellsignal.com/products/primary-antibodies/p53-1c12-mouse-mab/2524?site-search-type=Products&N=4294956287&Ntt=%232524s%29&fromPage=plp&_requestid=5815558

mouse anti-p53-HRP (Santa Cruz, sc-126)
https://www.scbt.com/p/p53-antibody-do-1

rabbit anti-phospho-P53 S15 (Cell Signaling, #9284)
https://www.cellsignal.com/products/primary-antibodies/phospho-p53-ser15-antibody/9284?site-search-type=Products&N=4294956287&Ntt=%239284%29&fromPage=plp&_requestid=5816166

mouse anti-p21 (Santa Cruz, sc-53870)
https://www.scbt.com/p/p21-antibody-sx118?requestFrom=search

rabbit anti-p16 (Santa Cruz, sc-1207)
rabbit anti-BAX (Cell Signaling, #2772) https://www.cellsignal.com/products/primary-antibodies/bax-antibody/2772?site-search-type=Products&N=4294956287&Ntt=%232772%29&fromPage=plp&_requestid=5816182

rabbit, anti-BAD (Cell Signaling, #9292)
https://www.cellsignal.com/products/primary-antibodies/bad-antibody/9292?site-search-type=Products&N=4294956287&Ntt=%239292%29&fromPage=plp&_requestid=5816207

rabbit anti-BCL2 (Santa Cruz, sc-492)
https://www.scbt.com/p/bcl-2-antibody-n-19?requestFrom=search

mouse anti-BCL-xL (Santa Cruz, sc-8392)
https://www.scbt.com/p/bcl-xl-antibody-h-5?requestFrom=search

rabbit, anti-α-Tubulin (Cell Signaling, #2125)
https://www.cellsignal.com/products/primary-antibodies/a-tubulin-11h10-rabbit-mab/2125?site-search-type=Products&N=4294956287&Ntt=%232125%29&fromPage=plp&_requestid=5816233

rabbit, anti-HDAC2 (Abcam, ab7029)

https://www.abcam.com/hdac2-antibody-ab7029.html

rabbit, anti-EGFR (Cell Signaling, #71655)
https://www.cellsignal.com/products/primary-antibodies/egf-receptor-d1p9c-rabbit-mab-mouse-preferred/71655?site-search-type=Products&N=4294956287&Ntt=%2371655%29&fromPage=plp&_requestid=5816267

mouse, anti-COX IV (Abcam, ab33985)
https://www.abcam.com/cox-iv-antibody-mabcam33985-mitochondrial-marker-ab33985.html

The RNASE4 antibody was validated in house for the use in Western Blot experiments using gene knockdown and gene overexpression samples. An example blot using Rnase4 knockdown samples is presented in Fig. 6e.

Eukaryotic cell lines

Policy information about cell lines

Cell line source(s)	Primary MEFs, mouse embryonic fibroblasts, were generated within the laboratory from genetically modified or unmodified mice. HEK-293T and IMR-90 cells were purchased from ATCC.
Authentication	PCR-based genotyping was used for cell identity authentication. Commercially available cell lines were not authenticated.
Mycoplasma contamination	All cultures were primary cultures that were used at early passage. They were not tested for mycoplasma contamination.
Commonly misidentified lines (See ICLAC register)	No commonly misidentified cell lines were used in this study.

Animals and other organisms

Policy information about studies involving animals; ARRIVE guidelines recommended for reporting animal research

Laboratory animals	Mice were used for generation of mouse embryonic fibroblasts as described in the methods section. MEFs were generated from embryos at embryonic day E13.5. The sex of embryos was not determined. Mice were maintained on a C57BL/6 NHsd genetic background. All mice were housed in a specific-pathogen-free (SPF) barrier environment with ad libitum access to food and water, 12-hour light and dark cycles, temperature between 68F-79F and humidity at ~70%.
Wild animals	Study did not involve wild animals.
Field-collected samples	No samples were collected from the field.
Ethics oversight	Experimental procedures involving laboratory mice were reviewed and approved by the Institutional Animal Care and Use Committee of the Mayo Clinic.

Note that full information on the approval of the study protocol must also be provided in the manuscript.
